# Chronic nicotine exposure attenuates the effects of Δ^9^‐tetrahydrocannabinol on anxiety‐related behavior and social interaction in adult male and female rats

**DOI:** 10.1002/brb3.1375

**Published:** 2019-10-03

**Authors:** Laurie A. Manwell, Tanya Miladinovic, Elana Raaphorst, Shadna Rana, Sarah Malecki, Paul E. Mallet

**Affiliations:** ^1^ Department of Psychology Wilfrid Laurier University Waterloo ON Canada

**Keywords:** anxiety‐related behavior, cross‐sensitization, delta‐9‐tetrahydrocannabinol, drug abuse, nicotine, social interaction

## Abstract

**Introduction:**

Anxiogenic and anxiolytic effects of cannabinoids are mediated by different mechanisms, including neural signaling via cannabinoid receptors (CBRs) and nicotinic cholinergic receptors (nAChRs). This study examined the effects of prior nicotine (the psychoactive component in tobacco) exposure on behavioral sensitivity to delta‐9‐tetrahydrocannabinol (THC; the psychoactive component of cannabis) challenge in animals.

**Methods:**

Male and female adult Sprague‐Dawley rats (*N* = 96) were injected daily with nicotine (1 mg/kg, i.p.) or vehicle for 14 days, followed by a 14‐day drug‐free period. On test day, rats were injected with THC (0.5, 2.0, or 5.0 mg/kg, i.p.) or vehicle and anxiety‐related behavior was assessed in the emergence (EM), elevated plus maze (EPM), and social interaction (SI) tests.

**Results:**

Chronic nicotine pretreatment attenuated some of the anxiogenic effects induced by THC challenge which can be summarized as follows: (a) THC dose‐dependently affected locomotor activity, exploratory behavior, and social interaction in the EM, EPM, and SI tests of unconditioned anxiety; (b) these effects of acute THC challenge were greater in females compared with males except for grooming a conspecific; (c) prior nicotine exposure attenuated the effects of acute THC challenge for locomotor activity in the EPM test; and (d) prior nicotine exposure attenuated the effects of THC challenge for direct but not indirect physical interaction in the SI tests.

**Conclusions:**

The ability of nicotine prior exposure to produce long‐lasting changes that alter the effects of acute THC administration suggests that chronic nicotine may induce neuroplastic changes that influence the subsequent response to novel THC exposure.

## Graphical abstract

Epidemiological studies suggest prior tobacco use increases the risk of cannabis use, but a causal relationship has not been established. This study demonstrated the effects of prior nicotine exposure on behavioral sensitivity to delta‐9‐tetrahydrocannabinol (THC) in male and female Sprague‐Dawley rats assessed in the emergence, elevated plus maze (EPM), and social interaction tests. Nicotine attenuated some of the anxiety‐producing effects of THC in both sex‐ and dose‐dependent manners. The ability of nicotine prior exposure to produce long‐lasting changes that alter the effects of acute THC administration suggests that chronic nicotine may induce neuroplastic changes that influence the subsequent response to novel THC exposure.

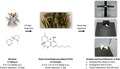

## 1 INTRODUCTION

The burden of disease attributable to substance use and the rates of comorbid tobacco and cannabis use are increasing, particularly for adolescents and young adults (Becker, Schaub, Gmel, & Haug, 2015; Degenhardt, Stockings, Patton, Hall, & Lynskey, 2016; Keyes, Hamilton, & Kandel, 2016; Patton, Coffey, Carlin, Sawyer, & Lynskey, 2005; Ramo, Liu, & Prochaska, 2012; Rubinstein, Rait, & Prochaska, 2014; Subramaniam, McGlade, & Yurgelun‐Todd, 2016). Tobacco use is the primary preventable cause of death with an estimated mortality rate of 5 to 6 million people per year globally (World Health Organization [WHO], 2008, 2009, 2013). Cannabis is the most widely used illicit drug with cannabis use disorders becoming increasingly prevalent (United Nations Office on Drugs and Crime [UNODC], 2015). Tobacco and cannabis use are highly comorbid, at rates of up to 90% in some studies, which has important implications for physical and psychosocial health (Agrawal, Budney, & Lynskey, 2012; Rabin & George, 2015). For example, the co‐occurrence of cannabis use disorder and nicotine dependence is associated with higher rates of psychiatric disorders, particularly anxiety disorders, bipolar disorder, and antisocial and schizotypal personality disorders (Peters, Schwartz, Wang, O'Grady, & Blanco, 2014). Daily or almost daily cannabis use is associated with anxiety disorders, particularly social anxiety (Feingold, Weiser, Rehm, & Lev‐Ran, 2016). Although tobacco and cannabis, and their respective psychoactive constituents, nicotine and tetrahydrocannabinol (THC), are reported to have anxiety‐alleviating effects, research in humans and animals also demonstrates their anxiogenic effects depending on the dose, timing, and route of administration and whether exposure is acute or chronic (Irvine, Cheeta, & File, 1999; Manwell, Charchoglyan, et al., 2014; Manwell, Ford, Ford, Matthews, Heipel, & Mallet, 2014; Manwell & Mallet, 2015; Morissette, Tull, Gulliver, Kamholz, & Zimering, 2007; Parrott, 1995; Pomerleau, Turk, & Fertig, 1984; Schramm‐Sapyta et al., 2007; Sethi et al., 1986; West & Hajek, 1997). Moreover, some of the adverse effects of THC, including intoxication, anxiety, and psychotic symptoms, are reduced by cannabidiol (CBD) also present in cannabis in varying amounts (Osborne, Solowij, & Weston‐Green, 2017; Solowij et al., 2019). A recent US national survey of tobacco use indicated that, although rates have decreased in the past decade, they remained constant for adults with one or more chronic health conditions and were greater in people with mental health comorbidities, specifically anxiety, depression, and substance abuse disorders (Stanton et al., 2016). The prevalence of comorbid tobacco and cannabis use is also greater in adolescents and young adults with mental illness and generally greater in males than in females (Hammerslag & Gulley, 2016; Ramo et al., 2012).

Epidemiological studies suggest that prior tobacco use increases the risk of cannabis use, but a causal relationship has not been well established. Evidence shows that illicit drug use (i.e., cannabis, cocaine, and heroin) is often preceded by tobacco and alcohol use, which supports the well‐known gateway hypothesis of drug use; however, cannabis use is a strong predictor for the onset and lifetime use of tobacco use, which supports the reverse gateway hypothesis (Agrawal et al., 2012; Becker et al., 2015; Fergusson, Boden, & Horwood, 2006; Kandel & Kandel, 2015; Keyes et al., 2016; Subramaniam et al., 2016; Wagner & Anthony, 2002). Thus, several neurobiological mechanisms for comorbid tobacco and cannabis have been proposed to explain the association, for example, synergistic mechanisms, involving central nervous system nicotinic acetylcholine receptors (nAChRs) and cannabinoid receptors (CBRs), and compensatory mechanisms, involving attenuation of withdrawal symptoms (Rabin & George, 2015; Subramaniam et al., 2016). Both of these mechanisms involve changes in the opposing physiological processes of reward and aversion learning which are mediated by neural circuits in the brain's mesolimbic dopaminergic system (MDS; Kandel & Kandel, 2015; Scherma et al., 2016). After nicotine exposure, neurons in the MDS become sensitized to the effects of other drugs, particularly THC and cocaine (Kandel & Kandel, 2015; Rabin & George, 2015; Subramaniam et al., 2016). Nicotine's effects on the endocannabinoid system include the release of endocannabinoids in the MDS, affecting dopamine levels and thus the rewarding effects of nicotine (González et al., 2002; Scherma et al., 2008); these effects can be blocked by administration of CBR antagonists, such as SR141716 (Cheer et al., 2007; Scherma et al., 2008). CBRs are also involved in the stress response: THC dose‐dependently elevates levels of stress hormones (e.g., corticosterone and adrenocorticotropic hormone [ACTH]; Schramm‐Sapyta et al., 2007), and downregulation of CBR‐mediated signaling induced by chronic stress is associated with impairments in behavioral flexibility and may play a role in repetitive behaviors notably observed in anxiety‐related neuropsychiatric disorders (Hill et al., 2005).

Animal models are advantageous in studying the role of nicotine and other drugs in the development of anxiety‐related disorders and impaired social behaviors, although more research is necessary to demonstrate the mechanisms involved (Le Foll, Ng, Di Ciano, & Trigo, 2015). Behavioral measures established to model unconditioned anxiety include the light–dark emergence test (EM), the elevated plus‐maze (EPM) test, and the social interaction (SI) test (Arrant, Schramm‐Sapyta, & Kuhn, 2013; Crawley, 1985; File, Cheeta, & Kenny, 2000; Le Foll et al., 2015; Pellow, Chopin, File, & Briley, 1985). The emergence test assesses competing approach‐avoidance motivations and clinically effective anxiolytics significantly increase exploration of the open‐lit compartment whereas anxiogenics reduce exploration (Arrant et al., 2013; Chauoloff, Durand, & Mormede, 1997; Crawley, 1985; Merlo Pich & Samanin, 1989). The EPM test also assesses approach‐avoidance conflict and clinically effective anxiolytic drugs, such as benzodiazepines (i.e., diazepam and chlordiazepam) and barbiturates (i.e., phenobarbitone), typically increase exploration of open arms without increasing exploration of closed arms (Montgomery, 1955; Pellow et al., 1985). In contrast, drugs with anxiogenic effects in humans (i.e., amphetamine, caffeine, yohimbine, and pentylenetetrazole) reduce the number of open arm entries and exploration time (Pellow et al., 1985). In comparison with animals confined to the closed arm, animals confined to the open arms show markedly elevated plasma corticosterone levels and more anxiety‐like behaviors (i.e., increased immobility, freezing behavior, and defecation; Pellow et al., 1985). The SI test assesses generalized anxiety behaviors in a social context measured by behaviors toward a conspecific, such as sniffing, following, grooming, and aggression; anxiolytic effects are generally inferred from increased social interaction, particularly in the absence of changes in locomotor activity reflecting nonspecific effects of a drug (File et al., 2000; Le Foll et al., 2015).

Evidence suggests the EM, EPM, and SI measures reflect different states of anxiety, which are potentially mediated by different neural mechanisms (Cheeta, Kenny, & File, 2000; File, 1992; File et al., 2000; File, Gonzalez, & Andrews, 1996; Genn, Tucci, Marco, Viveros, & File, 2004). By itself, nicotine induces anxiolytic or anxiogenic effects depending upon the dose and testing conditions (File, Cheeta, Irvine, Tucci, & Akthar, 2002; File, Kenny, & Ouagazzal, 1998; Irvine et al., 1999). Studies in male rodents show that acute or chronic exposure to high doses of cannabinoids tends to increase anxiogenic behaviors on these three tests, whereas low doses have anxiolytic effects (Berrendero & Maldonado, 2002; Genn, Tucci, Marco, Viveros, & File, 2003; Genn et al., 2004; Marco et al., 2004; Onaivi, Green, & Martin, 1990; O'Shea, McGregor, & Mallet, 2006; O'Shea, Singh, McGregor, & Mallet, 2004; Schramm‐Sapyta et al., 2007; Valjent, Mitchell, Besson, Caboche, & Maldonado, 2002). When coadministered, acute nicotine potentiates some of the cannabinoid‐induced responses on these measures (Valjent et al., 2002). Prior nicotine exposure also potentiates the aversive effects of high doses of THC, but not the rewarding effects of low doses of THC in place conditioning (Le Foll, Wiggins, & Goldberg, 2006), and is known to promote cross‐sensitization to other drugs including cocaine (Collins & Izenwasser, 2004; McQuown, Belluzzi, & Leslie, 2007), amphetamine (Collins, Montano, & Izenwasser, 2004), and morphine (Shippenberg, Heidbreder, & Lefevour, 1996). Several lines of evidence suggest that the anxiogenic and anxiolytic effects of cannabinoids are mediated by different mechanisms, including neural signaling via CBRs, nAChRs, serotonin (5‐HT1A) receptors, and opioid receptors (Berrendero & Maldonado, 2002; Marco et al., 2004; Valjent et al., 2002). Taken together, these findings suggest a physiological interaction between nicotine and THC in anxiety‐related behaviors.

Given this interaction, the objective of the current experiment was to determine whether chronic nicotine exposure induces neuroplastic changes, which in turn alter the acute effect of THC on anxiety. The present study was designed to examine the effects of prior chronic nicotine exposure on acute THC challenge in male and female adult rats. We hypothesized that the aversive effects of acute THC exposure would be attenuated (i.e., less anxiogenic) in male and female rats.

## 2 MATERIALS AND METHODS

### 2.1 Animals

Experimentally naïve adult CD IGS rats (*N* = 96 Charles River, QC, Canada) were used. At the beginning of the experiment, adult male (*n* = 50) and female (*n* = 46) rats weighed 225–250 g. Rats were fed standard rat chow (Harlan 8460) and water ad libitum and pair‐housed with a same‐sex, treatment‐matched partner in standard plastic shoebox cages (45 × 25 × 20 cm) in a colony room maintained at 21–22°C on a 12‐hr reverse light–dark cycle with light onset at 19:00 hr. All testing was conducted during the dark cycle. Experimental procedures followed Canadian Council on Animal Care Guidelines and were approved by the Wilfrid Laurier University Animal Care Committee. Rats were acclimatized to the colony room and handling procedures prior to experimentation.

### 2.2 Drugs

Nicotine ((–)‐nicotine tartrate salt; Sigma‐Aldrich) was dissolved in 0.9% NaCl, and the pH was adjusted to 7.0–7.4 with 0.1 M NaOH. Nicotine was administered (i.p.) at a dose of 1 mg/kg in a volume of 1 ml/kg body weight. This dose of nicotine was chosen to be within the range known to induce lasting changes in cannabinoid receptor density and activity (e.g., Werling, Collins Reed, Wade, & Izenwasser, 2009). Δ^9^‐Tetrahydrocannabinol (THC; THC Pharm GmbH) was dissolved in ethanol, mixed with a small quantity of TWEEN 80 (such that the final vehicle contained 1% TWEEN 80), and the ethanol was evaporated under a stream of nitrogen gas. THC was then suspended in 0.9% NaCl and injected (i.p.) in doses of 0.5, 2, or 5 mg/kg in a volume of 1 ml/kg. These multiple doses of THC for an acute challenge were chosen based upon previous studies showing the minimum doses for observable effects (e.g., locomotor, anxiety‐like behavior, conditioned preference, or aversion) up to doses that are less than those that begin to produce sedation effects (e.g., catalepsy; Le Foll et al., 2006; Schramm‐Sapyta et al., 2007; Werling et al., 2009).

### 2.3 Behavioral testing apparatus

#### 2.3.1 Emergence test

The emergence test was conducted in a dimly lit room illuminated by one 13 W compact fluorescent red lamp (5 Lux at apparatus level) within an apparatus consisting of a 120 × 120 × 45 cm white melamine arena with a black acrylonitrile butadiene styrene (ABS) floor and a 40 × 24 × 17 cm black melamine hide box. Rats were placed in the hide box at the beginning of the test period. Activity was recorded by a video camera mounted 225 cm above the apparatus, using the ANY‐maze video tracking software (Stoeling Co., 2010). Scored behaviors included latency to emerge from the hide box (s), time spent in the open field (s), and time mobile (s) to determine whether any changes in hide box latency may be related to altered locomotion.

#### 2.3.2 Elevated plus‐maze test

The EPM test consisted of two open (52 × 12 cm) and two closed (52 × 12 × 40 cm high) ultra‐high‐molecular‐weight polyethylene (UHMWPE) arms arranged in a cross‐elevated position, 53 cm above the room floor. The maze floor was constructed of black ABS. This task was conducted in a dimly lit room illuminated by one 13 W compact fluorescent red lamp (5 Lux at apparatus level) and activity was recorded by a camera mounted 140 cm above the apparatus, using ANY maze. Scored behaviors included number of entries to open and closed arms, time spent in open arms (s), and time mobile (s) which was used to quantify locomotor activity.

#### 2.3.3 Social interaction test

The social interaction test was conducted in a room dimly illuminated by white lights (37 Lux at apparatus level) and performed in an experimental chamber (61 × 26 × 40 cm) made of clear acrylic sides and top, and a black ABS floor. Animals were placed in the apparatus for 10 min with a treatment‐matched unfamiliar conspecific of approximately the same body weight. Activity was recorded by a video camera positioned 75 cm in front of the apparatus. An observer blind to group allocations manually scored trials using ODLog software (Macropod Software, http://www.macropodsoftware.com). Scored behaviors included time (s) spent sniffing, following, and grooming a conspecific, and time spent rearing as a measure of general locomotor activity.

### 2.4 Procedure

Rats (*n* = 12 per treatment group) were handled for seven consecutive days before the start of the experiment, after which half of the rats received chronic injections of nicotine and half received vehicle every 24 hr for 14 days. Following a two‐week washout period to ensure that tolerance and withdrawal effects would be reduced or absent (e.g., Irvine et al., 1999), rats were further divided into THC challenge groups that received either THC (0.5, 2.0, or 5.0 mg/kg, i.p.) or vehicle (1 ml/kg, i.p.) on test day (counterbalanced across nicotine pretreatment groups and males and females; see Table 1). Previous studies have shown that chronic nicotine exposure has lasting effects when rats are later challenged with an acute dose of THC (e.g., Werling et al., 2009). Rats were injected with THC or its vehicle, immediately returned to their home cage, and placed in either the emergence test or EPM 30 min later. Each rat was tested in the emergence test and EPM for 5 min per test. The order in which emergence and EPM testing were conducted was counterbalanced across groups. Following both tests, rats were placed in the social interaction test with an unfamiliar conspecific of the same sex and from the same treatment group for 10 min.

**Table 1:** Assignment of adult male and female rats to chronic nicotine pretreatment (1.0 mg/kg) and acute THC challenge conditions (0, 0.5, 2.0, and 5.0 mg/kg)

Nicotine pretreatment – THC challenge	Male	Female	Total
Veh‐Veh	7	5	12
Veh‐0.5 THC	6	6	12
Veh‐2.0 THC	6	6	12
Veh‐5.0 THC	6	6	12
Nic‐0.5 THC	6	6	12
Nic‐0.5 THC	6	6	12
Nic‐2.0 THC	6	6	12
Nic‐5.0 THC	7	5	12
Total	50	46	96

### 2.5 Data analysis

Data for all behavioral measures of the emergence test (time spent in open field, latency to exit the hide box, percentage of time spent mobile in the open field, mean locomotor speed, and open field entries), EPM test (number of open arm entries, time spent in open arms, time spent in closed arms, number of entries to closed arms, total time mobile, and mean locomotor speed), and social interaction test (sniffing, following, and grooming the other rat, and time spent rearing) were analyzed using three‐way 2 × 2 × 4 ANOVAs with sex (male vs. female), drug pretreatment condition (vehicle vs. 1.0 mg/kg nicotine), and acute drug challenge condition (vehicle vs. 0.5 mg/kg THC, 2.0 mg/kg THC, 5.0 mg/kg THC) as independent variables. Results were followed with one‐way ANOVAs and post hoc Dunnett's *t* (2‐sided) or Bonferroni's tests where warranted. For data with unequal variances (i.e., Levene's test significant) and/or sample sizes, Welch's *F* (*W*F) and Games‐Howell or Hochberg's GT2 post hoc tests were used where warranted (i.e., Field, 2009). The significance level was set at *p* < .05.

## 3 RESULTS

**Figure 1:**
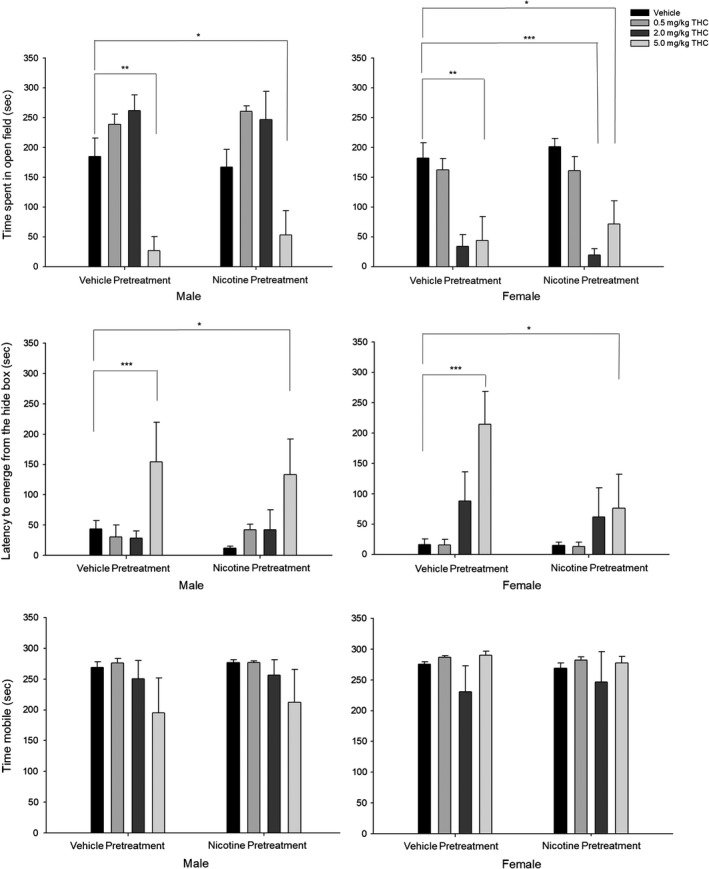
Light–dark emergence tests show anxiogenic effects of acute THC exposure. (Top panel) Time in open field (s). (Middle panel) Latency to emerge from the hide box (s). (Bottom panel) Time mobile (s). Behavioral data (means ± *SE*) for eight experimental conditions (*n* = 12/group; Veh‐Veh, Veh‐0.5THC, Veh‐2.0THC, Veh‐5.0THC, Nic‐Veh, Nic‐0.5THC, Nic‐2.0THC, and Nic‐5.0THC) in male and female adult rats. ANOVAs and Dunnett's *t* test (2‐sided): *p < .05 and **p < .01 and ***p < .001 compared to vehicle (Veh‐Veh)

**Figure 2:**
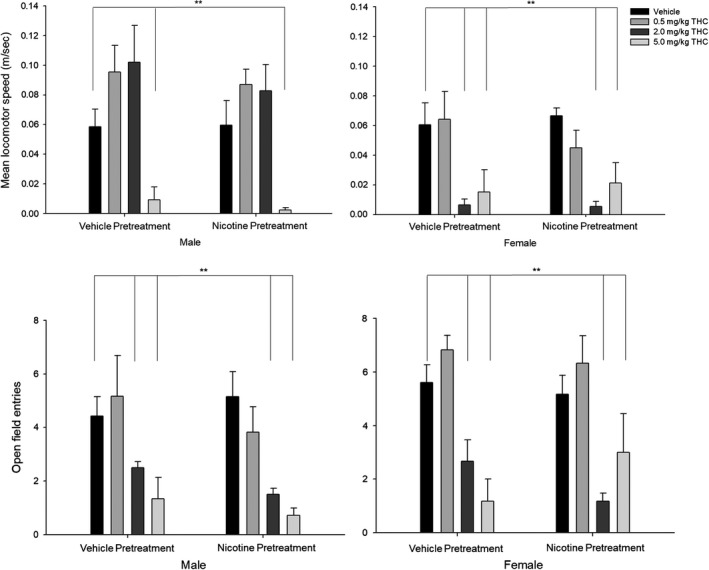
Light–dark emergence tests show anxiogenic effects of acute THC exposure. (Top panel) Mean locomotor speed (distance traveled (m)/time (s)). (Bottom panel) Open field entries. Behavioral data (means ± *SE*) for eight experimental conditions (*n* = 12/group; Veh‐Veh, Veh‐0.5THC, Veh‐2.0THC, Veh‐5.0THC, Nic‐Veh, Nic‐0.5THC, Nic‐2.0THC, and Nic‐5.0THC) in male and female adult rats. ANOVAs and Dunnett's *t* test (2‐sided): *p < .05 and **p < .01 and ***p < .001 compared to vehicle (Veh‐Veh)

**Figure 3:**
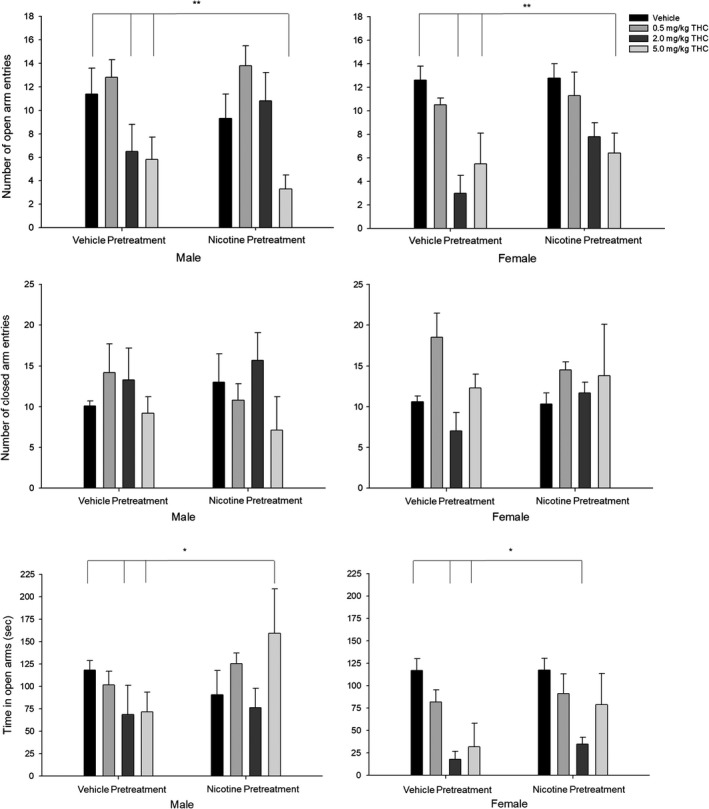
Elevated plus‐maze tests show anxiogenic effects of acute THC exposure. (Top panel) Number of open arm entries. (Middle panel) Number of closed arm entries. (Bottom panel) Time spent in open arms. Behavioral data (means ± *SE*) for the elevated plus‐maze test for eight experimental conditions (*n* = 12/group; Veh‐Veh, Veh‐0.5THC, Veh‐2.0THC, Veh‐5.0THC, Nic‐Veh, Nic‐0.5THC, Nic‐2.0THC, and Nic‐5.0THC) in male and female adult rats. ANOVAs and Dunnett's *t* test (2‐sided): *p < .05 and **p < .01 and ***p < .001 compared to vehicle (Veh‐Veh)

**Figure 4:**
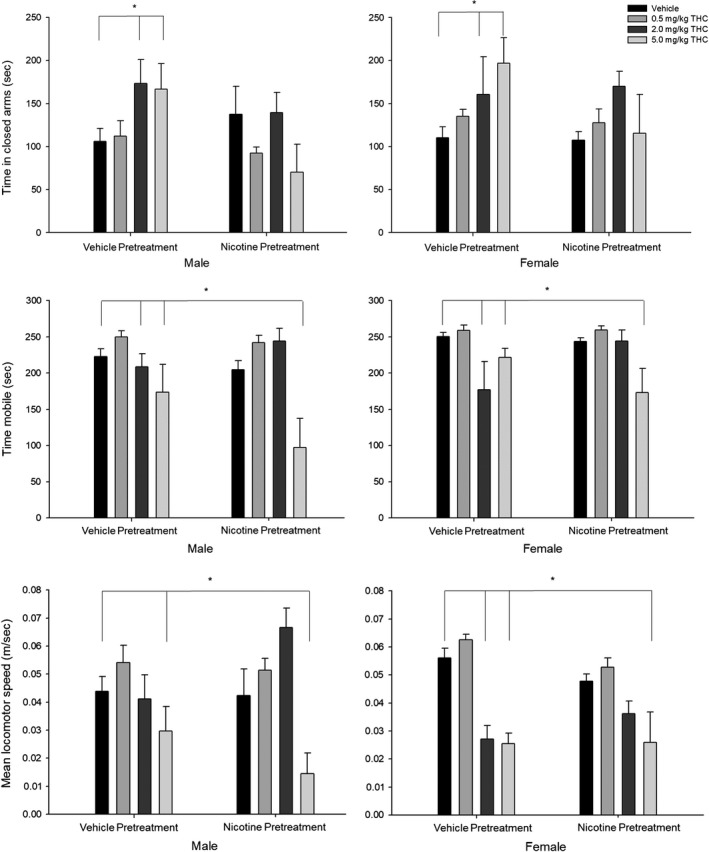
Elevated plus‐maze tests show anxiogenic and locomotor effects of acute THC exposure attenuated by prior nicotine exposure. (Top panel) Time spent in closed arms (s). (Middle panel) Time mobile (s). (Bottom panel) Mean locomotor speed (m/s). Behavioral data (means ± *SE*) for the light–dark emergence tests for eight experimental conditions (*n* = 12/group; Veh‐Veh, Veh‐0.5THC, Veh‐2.0THC, Veh‐5.0THC, Nic‐Veh, Nic‐0.5THC, Nic‐2.0THC, and Nic‐5.0THC) in male and female adult rats. ANOVAs and Dunnett's *t* test (2‐sided): *p < .05 and **p < .01 and ***p < .001 compared to vehicle (Veh‐Veh)

**Figure 5:**
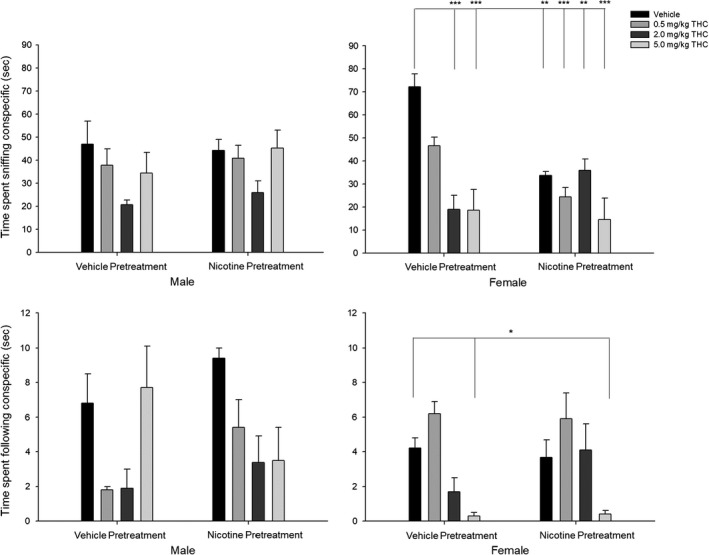
Social interaction tests show the effects of acute THC exposure and prior nicotine exposure on female rats. (Top panel) Time spent sniffing conspecific. (Bottom) Time spent following conspecific (s). Behavioral data (means ± *SE*) for the social interaction tests for eight experimental conditions (*n* = 12/group; Veh‐Veh, Veh‐0.5THC, Veh‐2.0THC, Veh‐5.0THC, Nic‐Veh, Nic‐0.5THC, Nic‐2.0THC, and Nic‐5.0THC) in male and female adult rats. ANOVAs and Dunnett's *t* test (2‐sided): *p < .05 and **p < .01 and ***p < .001 compared to vehicle (Veh‐Veh)

**Figure 6:**
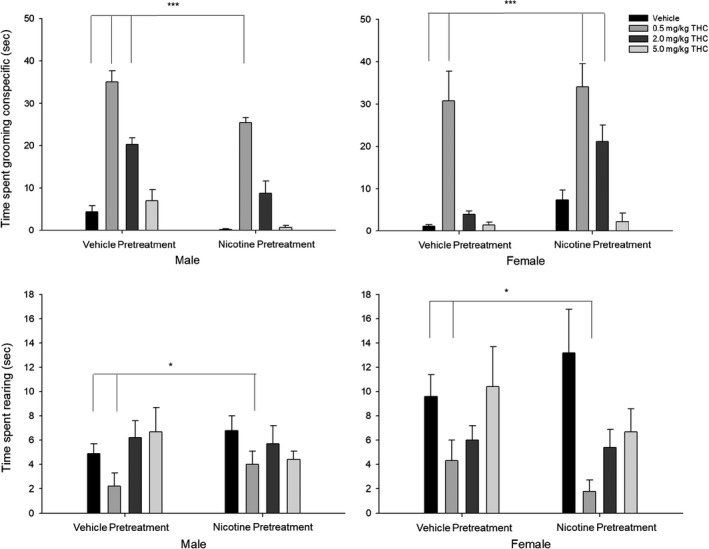
Social interaction tests show the effects of acute THC exposure and prior nicotine exposure on male and female Rats. (Top panel) Time spent grooming conspecific. (Bottom) Time spent rearing (s). Behavioral data (means ± *SE*) for the social interactions tests for eight experimental conditions (*n* = 12/group; Veh‐Veh, Veh‐0.5THC, Veh‐2.0THC, Veh‐5.0THC, Nic‐Veh, Nic‐0.5THC, Nic‐2.0THC, and Nic‐5.0THC) in male and female adult rats. ANOVAs and Dunnett's *t* test (2‐sided): *p < .05 and **p < .01 and ***p < .001 compared to vehicle (Veh‐Veh)

Results of the behavioral analyses with means (*M*) and standard errors (*SE*) are presented in Figures 1–2, 3–4, and 5–6 for the EM, EPM, and SI tests, respectively. There were no interaction or main effects of counterbalancing order for the EM, EPM, and SI tests (all *p*'s n.s.; see Table 2).

**Table 2:** Analysis of potential order effects for EM, EPM, and SI tests

		Order	Order × Sex	Order × Treatment	Order × Sex × Treatment
Emergence tests	Time in open field	*F* (1,67) = 0.839, *p* = .363	*F* (1,67) = 0.664, *p* = .418	*F* (6,67) = 0.896, *p* = .503	*F* (5,67) = 1.31, *p* = .270
	Latency to emerge	*F* (1,67) = 0.741, *p* = .391	*F* (1,67) = 0.030, *p* = .863	*F* (6,67) = 1.98, *p* = .081	*F* (5,67) = 0.138, *p* = .983
	Time mobile	*F* (1,67) = 0.502, *p* = .481	*F* (1,67) = 0.030, *p* = .863	*F* (6,67) = 1.31, *p* = .266	*F* (5,67) = 0.300, *p* = .911
	Mean locomotor speed	*F*(1,67) = 0.091, *p* = .764	*F* (1,67) = 0.387, *p* = .536	*F* (6,67) = 1.39, *p* = .229	*F* (5,67) = 0.392, *p* = .853
	Open field entries	*F* (1,67) = 0.163, *p* = .688	*F* (1,67) = 1.93, *p* = .170	*F* (6,67) = 1.11, *p* = .383	*F* (5,67) = 0.939, *p* = .462
Elevated plus maze	Number open arm entries	*F* (1,67) = 0.7.38, *p* = .393	*F* (1,67) = 1.02, *p* = .316	*F* (6,67) = 0.841, *p* = .543	*F* (5,67) = 0.374, *p* = .865
	Number closed arm entries	*F* (1,67) = 0.546, *p* = .463	*F* (1,67) = 0.014, *p* = .905	*F* (6,67) = 0.760, *p* = .604	*F* (5,67) = 0.837, *p* = .528
	Time spent in open arms	*F* (1,67) = 0.221, *p* = .640	*F* (1,67) = 0.002, *p* = .961	*F* (6,67) = 0.421, *p* = .863	*F* (5,67) = 1.2, *p* = .313
	Time spent in closed arms	*F* (1,67) = 0.084, *p* = .773	*F* (1,67) = 2.87, *p* = .094	*F* (6,67) = 0.872, *p* = .520	*F* (5,67) = 2.20., *p* = .064
	Time mobile	*F* (1,67) = 3.10, *p* = .082	*F* (1,67) = 0.525, *p* = .471	*F* (6,67) = 0.421, *p* = .862	*F* (5,67) = 0.420, *p* = .833
	Mean locomotor speed	*F* (1,67) = 2.10, *p* = .152	*F* (1,67) = 0.798, *p* = .375	*F* (6,67) = 1.05, *p* = .401	*F* (5,67) = 0.505, *p* = .772
Social interaction test	Sniffing conspecific	*F* (1,67) = 0.083, *p* = .775	*F* (1,67) = 0.699, *p* = .406	*F* (6,67) = 0.825, *p* = .555	*F* (5,67) = 1.22, *p* = .310
	Following conspecific	*F* (1,67) = 4.40, *p* = .083	*F* (1,67) = 0.934, *p* = .337	*F* (6,67) = 0.882, *p* = .513	*F* (5,67) = 0.573, *p* = .721
	Grooming conspecific	*F* (1,67) = 0.090, *p* = .765	*F* (1,67) = 1.46, *p* = .231	*F* (6,67) = 0.713, *p* = .640	*F* (5,67) = 1.01, *p* = .419
	Rearing (self)	*F* (1,67) = 0.057, *p* = .813	*F* (1,67) = 0.124, *p* = .726	*F* (6,67) = 1.12, *p* = .361	*F* (5,67) = 0.163, *p* = .975

### 3.1 Emergence test

#### 3.1.1 Time spent in open field

Analysis of the time (s) spent in the open field showed a significant interaction between Sex × THC challenge [*F*(3,80) = 16.33, *p* < .001, $\eta_{p}^{2}$ = 0.380] and significant main effects of Sex [*F*(1,80) = 24.19, *p* < .001, $\eta_{p}^{2}$ = 0.232] and THC challenge [*F*(1,80) = 23.39, *p* < .001, $\eta_{p}^{2}$ = 0.467] such that time in the open field was decreased for (a) female rats given the two highest doses of THC (2.0 and 5.0 mg/kg), (b) females compared with males, and (c) all rats given the highest dose of THC (5.0 mg/kg).

#### 3.1.2 Latency to emerge from the hide box

Analysis of the time (s) to emerge from the hide box showed only a significant main effect of THC challenge [*F*(1,80) = 9.86, *p* < .001, $\eta_{p}^{2}$ = 0.270] such that (a) latency to emerge from the hide box was increased for rats given only the highest dose of THC (5.0 mg/kg).

#### 3.1.3 Time mobile

Analysis of the total time mobile (s) showed that there were no significant differences across sex or treatment groups.

#### 3.1.4 Mean locomotor speed

Analysis of the mean locomotor speed (distance traveled (m)/time (s)) showed a significant interaction between Sex × THC challenge [*F*(3,80) = 11.853, *p* < .001, $\eta_{p}^{2}$ = 0.308] and significant main effects of Sex [*F*(1,80) = 16.18, *p* < .001, $\eta_{p}^{2}$ = 0.168] and THC challenge [*F*(1,80) = 16.03, *p* < .001, $\eta_{p}^{2}$ = 0.375] such that locomotor speed was decreased for (a) female rats given the two highest doses of THC (2.0 and 5.0 mg/kg), (b) females compared with males, and (c) all rats given the highest dose of THC (5.0 mg/kg).

#### 3.1.5 Open field entries

Analysis of the number of open field entries showed main effects of Sex [*F*(1,80) = 4.96, *p* < .05, $\eta_{p}^{2}$ = 0.058] and THC challenge [*F*(1,80) = 25.60, *p* < .001, $\eta_{p}^{2}$ = 0.490] such that open field entries were decreased for (a) males compared with females and (b) all rats given the two highest doses of THC (2.0 and 5.0 mg/kg).

### 3.2 Elevated plus maze

#### 3.2.1 Number of open arm entries

Analysis of the number of open arm entries showed a significant main effect of THC challenge [*F*(1,80) = 13.88, *p* < .001, $\eta_{p}^{2}$ = 0.342] such that the number of open arm entries was decreased for (a) all rats given the two highest doses of THC (2.0 and 5.0 mg/kg).

#### 3.2.2 Number of entries to closed arms

Analysis of the number of closed arm entries did not reveal any significant effects.

#### 3.2.3 Time spent in open arms

Analysis of the time spent in the open arms (s) main effects of Sex [*F*(1,80) = 6.35, *p* < .05, $\eta_{p}^{2}$ = 0.074] and THC challenge [*F*(1,80) = 4.88, *p* < .01, $\eta_{p}^{2}$ = 0.155] such that time spent in the open arms was decreased for (a) females compared with males, and (b) rats given the two highest doses of THC (2.0 and 5.0 mg/kg).

#### 3.2.4 Time spent in closed arms

Analysis of the time spent in the closed arms (s) showed a significant interaction between Nicotine pretreatment × THC challenge [*F*(3,80) = 3.00, *p* < 0.05, $\eta_{p}^{2}$ = 0.101] and main effects of Nicotine pretreatment [*F*(1,80) = 3.80, *p* < 0.05, $\eta_{p}^{2}$ = 0.045] and THC challenge [*F*(1,80) = 2.80, *p* < .05, $\eta_{p}^{2}$ = 0.095] such that time spent in the closed arms was increased for (a) rats given the two highest doses of THC (2.0 and 5.0 mg/kg) and (b) previous nicotine exposure abolished this effect of THC in all rats.

#### 3.2.5 Time mobile

Analysis of the time mobile (s) showed a significant interaction between Nicotine pretreatment × THC challenge [*F*(3,80) = 4.54, *p* < .01, $\eta_{p}^{2}$ = 0.145] significant main effect THC challenge [*F*(1,80) = 11.02, *p* < .001, $\eta_{p}^{2}$ = 0.292] such that the time mobile was (a) decreased for rats given the two highest doses of THC (2.0 and 5.0 mg/kg) and (b) this effect was attenuated for rats preexposed to nicotine and given the middle dose of THC (2.0 mg/kg).

#### 3.2.6 Mean locomotor speed

Analysis of the mean locomotor speed (distance traveled (m)/time (s)) showed a significant interaction between Sex × THC challenge [*F*(3,80) = 5.15, *p* < .01, $\eta_{p}^{2}$ = 0.162], a significant interaction between Nicotine pretreatment × THC challenge [*F*(3,80) = 3.53, *p* < .05, $\eta_{p}^{2}$ = 0.117], and a significant main effect of THC challenge [*F*(1,80) = 18.01, *p* < .001, $\eta_{p}^{2}$ = 0.403] such that locomotor speed was decreased for (a) female rats given the two highest doses of THC (2.0 and 5.0 mg/kg) and (b) all rats given the highest dose of THC (5.0 mg/kg), and (c) nicotine preexposure attenuated this effect for rats given the middle dose of THC (2.0 mg/kg).

### 3.3 Social interaction test

#### 3.3.1 Time spent sniffing conspecific

Analysis of the time (s) spent sniffing a conspecific showed significant interaction between Nicotine pretreatment × THC challenge [*F*(3,80) = 3.740, *p* < .05, $\eta_{p}^{2}$ = 0.123], between Sex × Nicotine pretreatment [*F*(3,80) = 4.881, *p* < .05, $\eta_{p}^{2}$ = 0.058], and between Sex × THC challenge [*F*(3,80) = 3.55, *p* < .05, $\eta_{p}^{2}$ = 0.118], and a significant main effect of THC challenge [*F*(1,80) = 8.80, *p* < .001, $\eta_{p}^{2}$ = 0.248] such that time spent sniffing was decreased for (a) female rats preexposed to vehicle and the two highest doses of THC (2.0 and 5.0 mg/kg), and (b) female rats preexposed to nicotine, and (c) all rats preexposed to vehicle and the two highest doses of THC (2.0 and 5.0 mg/kg).

#### 3.3.2 Time spent following conspecific

Analysis of the time (s) spent following a conspecific showed a significant interaction between Sex × THC challenge [*F*(3,80) = 7.30, *p* < .001, $\eta_{p}^{2}$ = 0.215] and significant main effects of Sex [*F*(1,80) = 6.05, *p* < .05, $\eta_{p}^{2}$ = 0.070] and THC challenge [*F*(1,80) = 5.54, *p* < .01, $\eta_{p}^{2}$ = 0.172] such that time spent following the conspecific was decreased for (a) female rats given the highest dose of THC (5.0 mg/kg) and (b) females than males.

#### 3.3.3 Time spent grooming conspecific

Analysis of the time (s) spent grooming a conspecific showed a significant interaction between Sex × Nicotine pretreatment × THC challenge [*F*(1,80) = 2.72, *p* < .05, $\eta_{p}^{2}$ = 0.093], between Sex × Nicotine pretreatment [*F*(3,80) = 25.75, *p* < .001, $\eta_{p}^{2}$ = 0.2443], and a significant main effect of THC challenge [*F*(1,80) = 84.13, *p* < .001, $\eta_{p}^{2}$ = 0.759] such that time spent grooming the conspecific was increased for (a) males preexposed to vehicle and given the two lowest doses of THC (0.5 and 2.0 mg/kg), (b) males preexposed to nicotine and given the lowest dose of THC (0.5 mg/kg), (c) females preexposed to vehicle and given the lowest dose of THC (0.5 mg/kg), and (d) females preexposed to nicotine and given the two lowest doses of THC (0.5 and 2.0 mg/kg).

#### 3.3.4 Time spent rearing

Analysis of the time (s) spent rearing showed significant main effects of Sex [*F*(1,80) = 5.54, *p* < .05, $\eta_{p}^{2}$ = 0.065] and THC challenge [*F*(1,80) = 7.13, *p* < .001, $\eta_{p}^{2}$ = 0.211] such that time spent rearing was decreased for (a) males compared with females and (b) rats given the lowest dose of THC (0.5 mg/kg).

## 4 DISCUSSION

The present study demonstrates that nicotine reduced sensitivity to some of the anxiogenic effects of THC. These results show that acute THC induced anxiety‐like behavior in adult rats, which was generally greater in females than in males. Prior chronic nicotine exposure attenuated some of the anxiogenic effects of acute THC without producing lasting effects on its own. Dose‐ and task‐specific effects can be summarized as follows: (a) THC dose‐dependently affected locomotor activity, exploratory behavior, and social interaction in the EM, EPM, and SI tests of unconditioned anxiety; (b) these effects of acute THC challenge were greater in females compared with males except for grooming a conspecific; (c) prior nicotine exposure attenuated the effects of acute THC challenge for locomotor activity in the EPM test; and (d) prior nicotine exposure attenuated the effects of THC challenge for direct but not indirect physical interaction in the SI tests. This evidence of nicotine's potential to attenuate some of the aversive effects of THC provides support for the hypothesis of a functional link between the cholinergic and cannabinoid systems that may underlie increased risk of cannabis use arising from prior tobacco use.

### 4.1 Novelty of anxiety‐related behavioral findings

Results of the current study add to the body of research demonstrating the broad anxiogenic effects of high doses of THC, which are influenced by sex, and present new findings demonstrating that nicotine has long‐lasting effects on the endocannabinoid system that moderate a range of anxiety‐related behaviors in male and female rats. Results of the EM, EPM, and SI tests demonstrated that prior exposure to chronic nicotine alone did not affect anxiety‐related behaviors but did attenuate some of the anxiogenic behaviors that were dose‐dependently induced by acute THC in both male and female adult rats.

First, the EM and EPM test results are highly consistent with previous studies demonstrating anxiogenic effects of THC at similar doses in male rodents (Schramm‐Sapyta et al., 2007; Valjent et al., 2002). Specifically, acute THC dose‐dependently produces anxiogenic responses in both the EM and EPM tests with some locomotor‐suppressing effects in male rats (0.5 to 2.5 mg/kg; Schramm‐Sapyta et al., 2007) and in the EM test in male mice (5 mg/kg; Valjent et al., 2002). In the EPM test, the CB1 receptor agonist CP 55,940 produces anxiogenic and anxiolytic responses at high and low doses, respectively, in male rats (Marco et al., 2004).

Second, the SI test results are also consistent with previous findings that acute exposure to CB1 receptor agonists reduces social interaction in male rats, including THC (Malone, Jongejan, & Taylor, 2009; van Ree, Niesink, & Nir, 1984), CP 55,940 (Genn et al., 2004), and WIN 55212‐2 (Trezza & Vanderschuren, 2008a,2008b) even inducing conditioned anxiety in subsequent drug‐free tests (Genn et al., 2004). Chronic exposure to CB1 agonists also reduces social interaction in drug‐free tests in male adolescent and adult rats (O'Shea et al., 2006) and female adolescent rats but not female adult rats (O'Shea et al., 2004). Grooming behavior (of a conspecific) was the only measure in the current study to suggest an anxiolytic effect of low dose THC; however, the effect was moderated by both sex and nicotine exposure. Self‐grooming and social‐grooming behaviors recruit neighboring, but functionally dissociable, inhibitory and excitatory neurons in the medial amygdala that act antagonistically (Hong, Kim, & Anderson, 2014; Kalueff et al., 2016), controlling the induction and suppression of social and asocial behaviors (Hong et al., 2014). Opposing behaviors could be triggered by activation of CB1 receptors via THC, in neighboring amygdala neurons, potentially explaining the simultaneous occurrence of decreased sniffing, following, and rearing, but increased grooming behaviors in the present study. Similarly, rearing is considered a measure of exploratory and/or escape behavior in rats (Lever, Burton, & O'Keefe, 2006) and could be mediated by similar mechanisms in the amygdala (Hong et al., 2014), as suggested by research showing increased dopaminergic activity is associated with decreased rearing induced by THC (Hernández‐Tristán, Arévalo, Canals, & Leret, 2000).

Third, measures of locomotor activity in the EM, EPM, and SI tests showed that THC had no effect, or a locomotor‐suppressing effect, with some sex differences and interaction with nicotine observed. Previous studies show locomotor‐suppressing effects of THC in rats (Allen, McGregor, Hunt, Singh, & Mallet, 2003; McGregor, Arnold, Weber, Topple, & Hunt, 1998; Schramm‐Sapyta et al., 2007; Tseng & Craft, 2001) which are greater in adults than adolescents (Schramm‐Sapyta et al., 2007) and in females than males (Tseng & Craft, 2001). Nicotine has been reported to potentiate various effects of high doses of THC (e.g., hypothermia, hypolocomotion, antinociception, tolerance, and precipitated withdrawal) and low doses of THC (e.g., anxiolytic responses and conditioned place preference) in male mice (Valjent et al., 2002). Although it is possible the reduction in anxiety‐like behaviors could be a by‐product of a reduced ability or desire to move, this is unlikely because overall mobility was not generally affected or it was attenuated by prior nicotine exposure.

Thus, the present study extends previous findings in adult male rats and mice to demonstrate that THC also induces anxiogenic behaviors in adult female rats, which is greater than in males, and the anxiogenic effects of THC are lessened by prior nicotine exposure for both male and female rats, although females are also more sensitive to the effects of nicotine. The present results are also consistent with previous findings that other behavioral effects induced by THC (e.g., antinociception and catalepsy) are greater in female than in male rats (Tseng & Craft, 2001), that prior nicotine exposure can alter other behavioral effects induced by THC (Trauth, Seidler, & Slotkin, 2000), and that sensitization to nicotine varies depending upon sex and age in rats (Collins & Izenwasser, 2004; Collins, Montano, et al., 2004; Faraday, Elliott, & Grunberg, 2001; Schochet, Kelley, & Landry, 2004). The mediation of THC‐induced effects by sex and prior nicotine exposure in the current study is also consistent with reports in animals and in humans (Fattore, Altea, & Fratta, 2008; Subramaniam et al., 2016), for example, strong associations between anxiety and reduced social functioning in cannabis users (Feingold et al., 2016), greater risk of anxiety‐related disorders in younger female cannabis users (Patton et al., 2002), and higher rates of anxiety‐related disorders in individuals reporting cannabis and nicotine dependence (Peters et al., 2014).

### 4.2 Potential mechanisms involved

Several potential neurobiological mechanisms may underlie nicotine's mediation of different types of THC‐induced anxiogenic behaviors in male and female rats. First, nicotine and THC administered alone induce similar pharmacological effects (Ahsan et al., 2014; Howlett et al., 1990; Jackson et al., 2010; Justinová, Goldberg, Heishman, & Tanda, 2005; Lichtman, Cook, & Martin, 1996; Sañudo‐Peña, Romero, Seale, Fernandez‐Ruiz, & Walker, 2000; Scherma et al., 2016) which are dose‐dependent and biphasic, typically producing anxiolytic effects at low doses and anxiogenic effects at high doses (Brioni et al., 1994; Cheeta, Irvine, Kenny, & File, 2001; Olausson, Akesson, Engel, & Söderpalm, 2001; Ouagazzal, Kenny, & File, 1999; Patel & Hillard, 2006; Viveros, Marco, & File, 2005). Second, when coadministered, nicotine mediates THC‐induced behavioral effects, which are potentiated (Balerio, Aso, Berrendero, Murta, & Maldonado, 2004; Balerio, Aso, & Maldonado, 2006; Le Foll et al., 2006; Pryor, Larsen, Husain, & Braude, 1978; Scherma et al., 2012, 2016; Valjent et al., 2002) or attenuated (Le Foll et al., 2006), even when administered at subthreshold levels (Valjent et al., 2002). Acute nicotine potentiates a range of stress‐related responses induced by THC, including unconditioned anxiogenic and anxiolytic responses, conditioned place preference, antinociception, hypolocomotion, and hypothermia, which involve activation of neural circuits in the MDS, including the amygdala and prefrontal cortex (Valjent et al., 2002), brain regions involved in emotional regulation, and expressing high densities of CBRs and nAChRs (Viveros et al., 2005; Watkins, Koob, & Markou, 2000). Third, the degree of nAChR activation by nicotine appears to affect downstream regulation of neurotransmitters in the MDS differently (Watkins et al., 2000). Acute nicotine briefly stimulates nAChRs (Corringer et al., 1998), activating dopaminergic and serotonergic neurons (Bang & Commons, 2011; Nisell, Nomikos, & Svensson, 1994), after which nAChRs become transiently desensitized (Corringer et al., 1998). Consequently, chronic nicotine upregulates nAChRs (Wonnacott, 1997) across several brain regions (Collins, Wade, Ledon, & Izenwasser, 2004; Doura, Gold, Keller, & Perry, 2008; Slotkin, Cousins, & Seidler, 2004; Trauth, Seidler, McCook, & Slotkin, 1999) leading to increased dopamine (Carboni, Bortone, Giua, & Chiara, 2000) and AEA and 2‐AG levels in the brain (González et al., 2002; Scherma et al., 2008). However, age of first exposure to nicotine greatly affects the distribution and density of nAChRs (Doura et al., 2008). Specifically, nAChR subtype α4β2* receptors are expressed more abundantly in drug‐naïve adolescent rats than in adult rats (Doura et al., 2008) and chronic nicotine exposure upregulates nAChRs in greater numbers across more brain regions in adult rats than in adolescent rats (Collins, Wade, et al., 2004; Doura et al., 2008; Slotkin et al., 2004; Trauth et al., 1999). Fourth, during nicotine abstinence, there is a period of recovery of nAChR function (Dani & Heineman, 1996; Koob, Sanna, & Bloom, 1998) that coincides with the somatic and motivational effects of withdrawal, which peak at 10–16 hr and return to baseline around 96 hr after the last nicotine exposure (Shoaib & Bizarro, 2005). Symptoms of nicotine withdrawal are associated with decreased dopaminergic function in the MDS, particularly the amygdala, and thus, it is hypothesized that protracted abstinence involves neuroreadaptation of dopaminergic function in the amygdala that affects the stress response, mood, and anxiety levels, essentially creating a new “hedonic set point” (Koob, 1996; Koob & Le Moal, 1997; Watkins et al., 2000) and potentially contributing to greater tolerance of the anxiogenic effects induced by high doses of THC (Valjent et al., 2002). THC is known to activate the HPA axis, increasing stress hormone levels and prolonging their circulation in the bloodstream (Patel, Cravatt, & Hillard, 2005; Schramm‐Sapyta et al., 2007), which likely contributes to its anxiogenic effects at high doses. Thus, neuroreadaptation of dopaminergic function in the MDS, particularly in the amygdala, after chronic nicotine exposure, may have led to a diminished stress response to high doses of THC that induced anxiogenic effects in male and female rats in the current experiment. Fifth, sex‐related differences in anxiogenic responses observed in the current study may be related to hormonal levels in female rats; higher levels of oestradiol in cycling female rats are associated with decreased CB1 receptor densities in the prefrontal cortex and amygdala and reduced motor activity and impaired social interaction (Castelli et al., 2014). Sex differences are also found in the effects of chronic nicotine on different types of locomotor activity, such as horizontal versus vertical locomotor activity, and in the moderating effects of stress (Faraday, O'Donoghue, & Grunberg, 2003). These findings are consistent with some of the sex differences reported in the association between comorbid THC and nicotine use and anxiety‐related disorders in humans (Hammerslag & Gulley, 2016; Ramo et al., 2012).

### 4.3 Implications and future research

Increasing trends worldwide toward cannabis legalization are associated with higher rates of tobacco and cannabis co‐use, and co‐use is a significant predictor of nicotine dependence for adolescents and adults (Wang, Ramo, Lisha, & Cataldo, 2016). This increase coincides with chronic cannabis users reporting smoking cannabis primarily to relieve symptoms of both physical conditions (e.g., sleep disturbances, pain, and concentration problems) and psychological conditions (e.g., anxiety, stress, and depression) rather than for merely recreational use (Bottorff, Johnson, Moffatt, & Mulvogue, 2009; Hyman & Sinha, 2009; Temple, Driver, & Brown, 2014). Addressing comorbid drug use and psychiatric symptoms requires a better understanding of the biological mechanisms that link the cannabinoid and cholinergic systems in the brain, specifically areas of structural and functional overlap between their neurotransmitters and receptors in mediating the rewarding and aversive effects of nicotine and THC. The present study's results provide additional support for theories of tobacco and cannabis co‐use focusing on compensatory effects, specifically, that nicotine and THC attenuate each other's negative effects and aversive states (reviewed in Rabin & George, 2015). For example, tobacco and cannabis can be used to mitigate each other's withdrawal symptoms (e.g., dysphoria, cravings, irritability, and sleep disturbances) and cognitive and affective impairments (e.g., THC can produce both euphoria and paranoia and impairs learning and memory; nicotine increases arousal and improves concentration and cognition; Rabin & George, 2015). Pharmacological treatments targeting the cannabinoid and/or cholinergic systems may prove beneficial in weakening the psychological and neurobiological associations between tobacco and cannabis and thus reducing their co‐use.

Future research could focus on other common mechanisms underlying tobacco and cannabis use, including conventional routes of administration (via smoking), similar environmental influences, particularly social stressors, and shared genetic factors (Agrawal et al., 2012; Rabin & George, 2015). For example, future studies could compare the effects of tobacco smoke and cannabis smoke on anxiety‐related behavior and social interaction adult and adolescent rats. Indeed, in a limited number of studies, extracts from abused drugs (e.g., THC from marijuana, salvinorin A from dried *Salvia* leaves, and toluene from industrial chemicals) have been shown to produce different effects on behavior and memory depending upon the route of administration, specifically whether they are inhaled or injected (Benignus, Muller, Barton, & Bittikofer, 1984; Fá et al., 2000; Manwell, Charchoglyan, et al., 2014; Manwell, Ford, et al., 2014; Manwell & Mallet, 2015; Manwell et al., 2015; Naef, Russman, Petersen‐Felix, & Brenneisen, 2004; Niyuhire, Varvel, Martin, & Lichtman, 2007; Perit et al., 2012). Future studies could also evaluate the potential of other agents to attenuate the anxiety‐ and abuse‐related behavioral effects of both nicotine and THC, including cannabidiol, a major nonpsychoactive constituent of marijuana (Viveros et al., 2005), fatty acid amide (FAAH) inhibitors such as URB597 that prevent the degradation of natural endocannabinoids (Scherma et al., 2012), and D3 antagonists that target dopaminergic neurons activated by nAChR and CBR signaling (Le Foll, Goldberg, & Sokoloff, 2005; Le Foll, Schwartz, & Sokoloff, 2000; Le Foll, Sokoloff, Stark, & Goldberg, 2005; Pak et al., 2006).

## 5 CONCLUSIONS

The current study demonstrates that there are important differences in THC‐induced anxiety‐related behavior that are sex‐ and dose‐dependent and attenuated by prior nicotine exposure. Our results provide evidence in support of a broad anxiogenic profile for high doses of THC in male rodents, extend those findings to show similar but augmented responses in female rodents, and present new data demonstrating that these behavioral responses can be modified by prior exposure to nicotine. The ability of nicotine preexposure to produce long‐lasting changes that alter the effects of acute THC administration suggests that chronic nicotine may induce neuroplastic changes that contribute to both anxiety‐related disorders and cannabis use. These findings contribute to the existing literature on functional interactions between the cholinergic and endocannabinoid systems in the MDS and help explain the strong association between comorbid nicotine and cannabis use and increased risk of stress‐ and anxiety‐related disorders in epidemiological studies.

## CONFLICT OF INTERESTS

None declared.

## AUTHOR CONTRIBUTIONS

PEM designed the project, provided the laboratory and resources, and supervised the entire project. TM, ER, and SM conducted the experiments and collected the data. LAM analyzed and interpreted the data and wrote the manuscript. LAM is the guarantor of the manuscript.

## Data Availability

The data that support the findings of this study are available from the corresponding author upon reasonable request.
